# Pre-surgical Provisional Prosthesis for Immediate Non-occlusal-loaded Flapless Implant

**DOI:** 10.7759/cureus.1345

**Published:** 2017-06-13

**Authors:** Athiban Inbarajan, Fathima Banu, Padmanabhan TV, Anand Kumar, Madhan Seenivasan

**Affiliations:** 1 Department of Prosthodontics, Faculty of Dental Sciences, Sri Ramachandra University, Porur, Chennai, India

**Keywords:** flapless surgery, immediate non-occlusal loading, provisional

## Abstract

A 49-year-old patient reported for immediate replacement of missing maxillary anterior teeth with implant-retained prosthesis. Elevation of flap alters the mucosal level, causes discomfort, and delays the restorative procedure. To maintain the esthetics, flapless surgery was planned. Since placement of an implant is pre-planned in a predetermined site, fabrication of the prostheses before commencement of the surgery, especially when replacing the teeth in the anterior region, could be a viable option. This case report explains the method of fabrication of the provisional restoration for flapless surgery in the presurgical phase. The technique would avoid any micromotion and implant instability caused due to abutment preparation and impression procedure postsurgically.

## Introduction

Full thickness mucoperiosteal flap elevation was practiced during the early period of implant surgical treatment to visualize the bone quality and quantity [1]. Flap elevation during the surgical procedure leads to alteration of the mucosal level, discomfort, and delayed healing. Studies reveal that full thickness flap elevation leads to exposure of the periosteal bone to external environment, which would trigger postsurgical tissue loss, and hence the resorption of bone [2]. In such a situation, maintaining esthetics would be critical, especially when replacing an anterior tooth. To overcome the disadvantage of mucoperiosteal flap elevation, a flapless approach was introduced, which enabled the placement of an implant without surgical exposure of the underlying bone, while preserving circulation and esthetics of soft tissue contours [3]. This was a drastic revolution from the concept of flap exposure and isolating implants placed into fresh extraction sockets with a barrier membrane and a primary flap closure [4]. With the introduction of cone beam computed tomography (CBCT), three-dimensional assessment of probable implant sites facilitate the use of flapless surgery for an implant placement [5]. Since placement of an implant is pre-planned in a predetermined site, fabrication of the prostheses before commencement of the surgery, especially when replacing the teeth in the anterior region, could be a viable option. There are literature evidences that show that soft tissue levels are more esthetic in the anterior zone when implants are torqued into freshly extracted sockets compared with a delayed strategy [6]. The technique explained in the present case report explains the presurgical fabrication of the provisional restoration for flapless surgery using a surgical stent as a guide.

## Technical report

**Figure 1:**
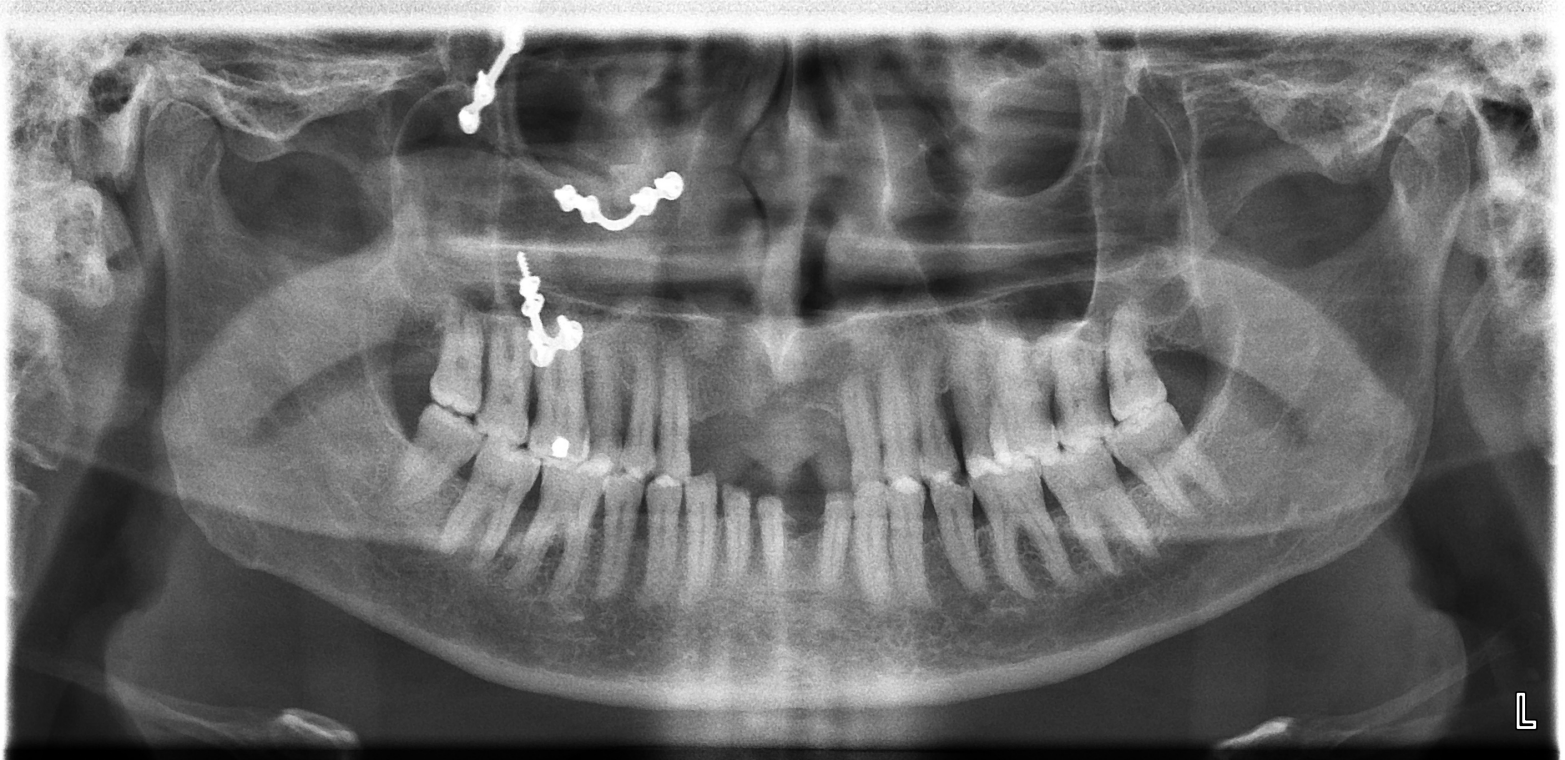
Radiographic investigation

A 49-year-old patient reported to the department of prosthodontics with the chief complaint of replacing missing maxillary anterior teeth. On examination, the patient had the following teeth missing: 11, 12, 21, 22, and 31. Radiographic imaging, CBCT investigation, and diagnostic impression (Figure 1) were made to evaluate the options for the replacement of the missing teeth.

**Figure 2:**
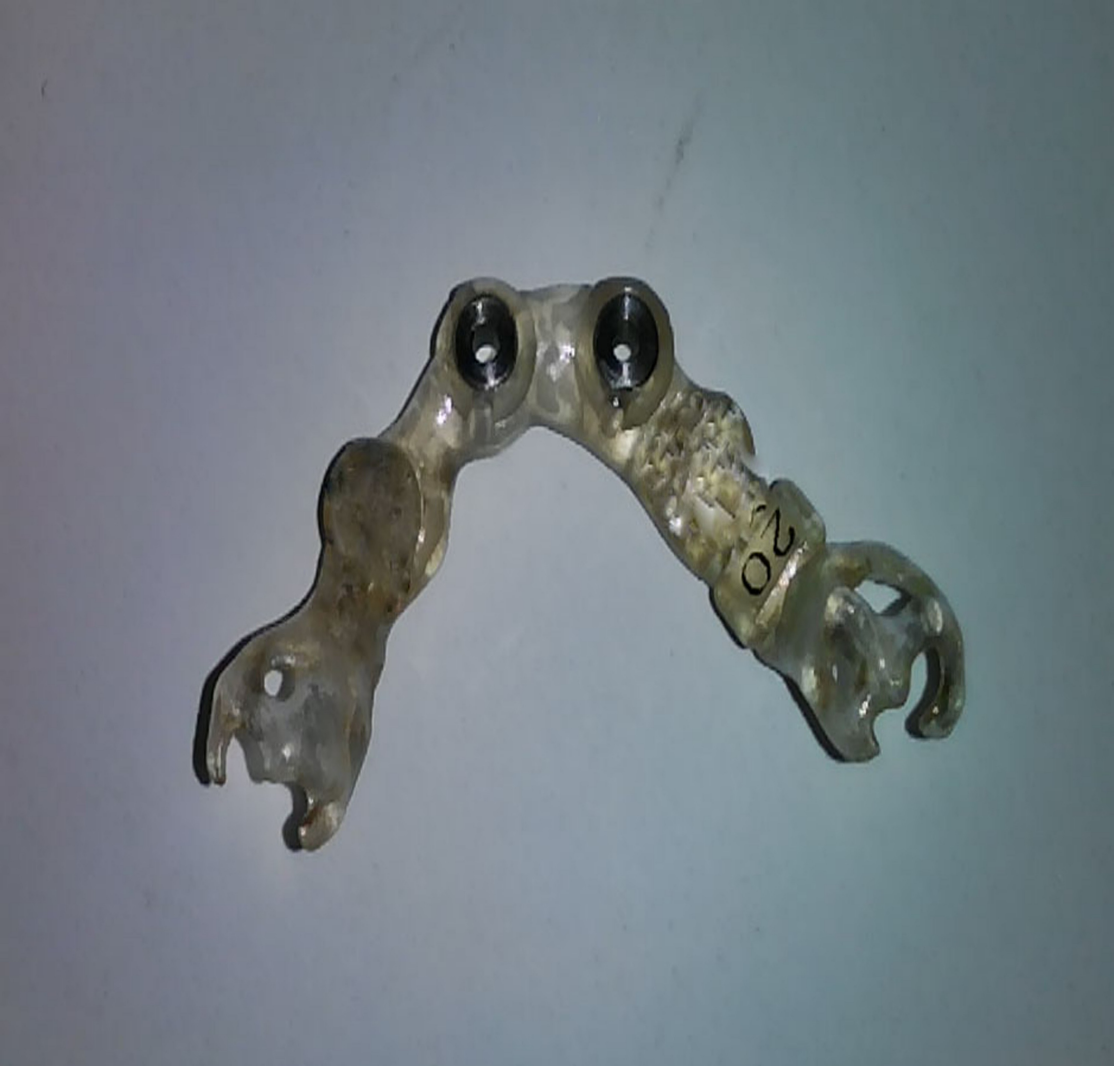
Surgical template fabricated using CBCT

Flapless implant surgery was planned with immediate non-occlusal loading. Evaluation of the angulation and width of the bone was done using CBCT and a surgical stent was fabricated (Figure 2).

**Figure 3:**
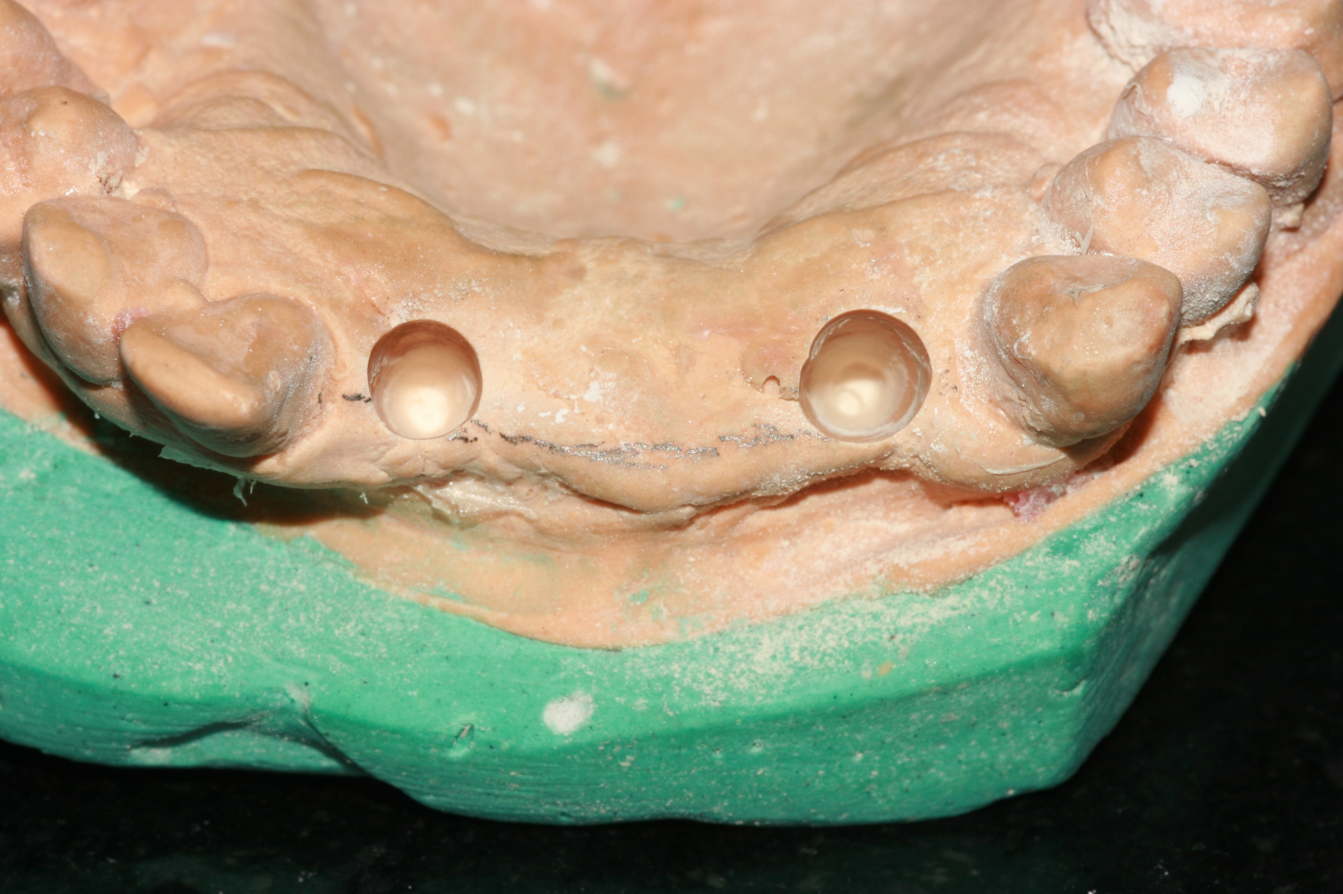
Mock surgery in the cast for determining implant position

Mock surgery was performed in the diagnostic cast determining the implant position with respect to 12 and 22 (Figure 3).

**Figure 4:**
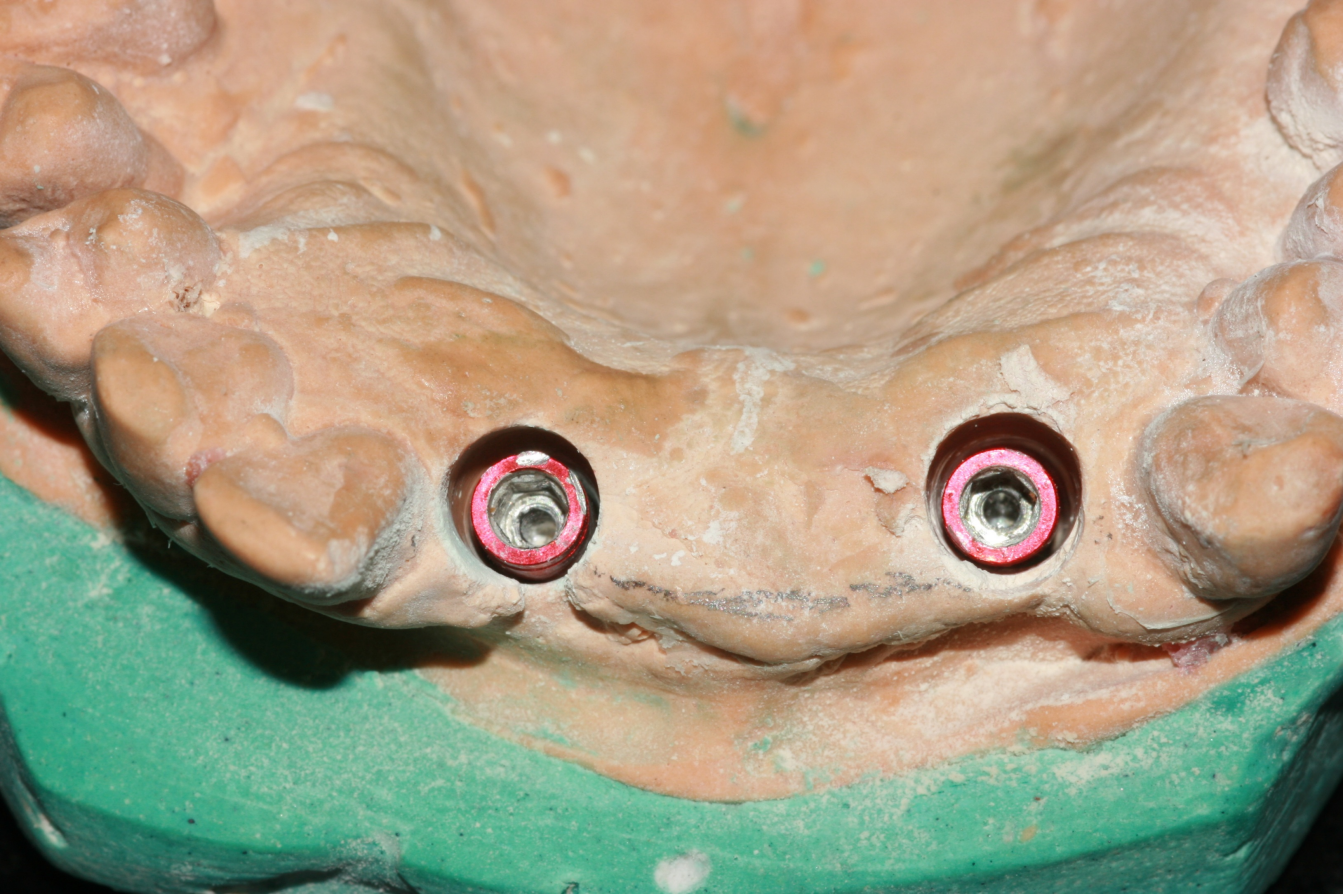
Positioning of the lab analogs

The lab analogs were placed in the mock osteotomy site (Figure 4).

**Figure 5:**
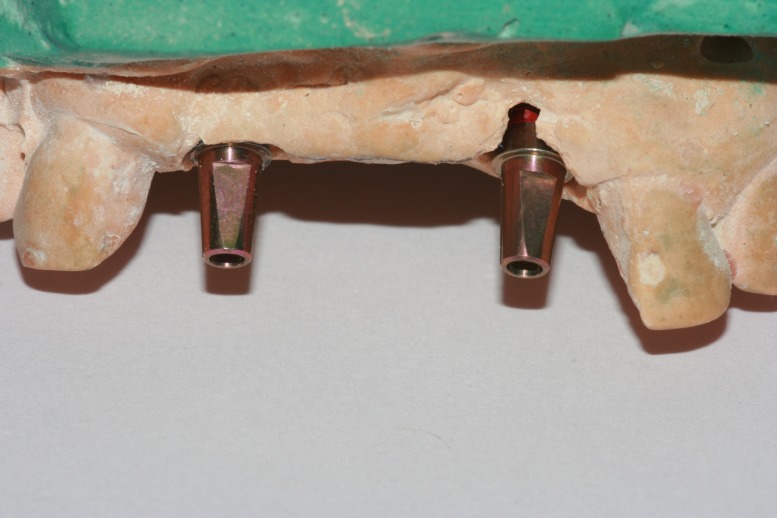
Abutment preparation

Abutments were screwed into the lab analogs, and preparation of the abutment was completed (Figure 5).

**Figure 6:**
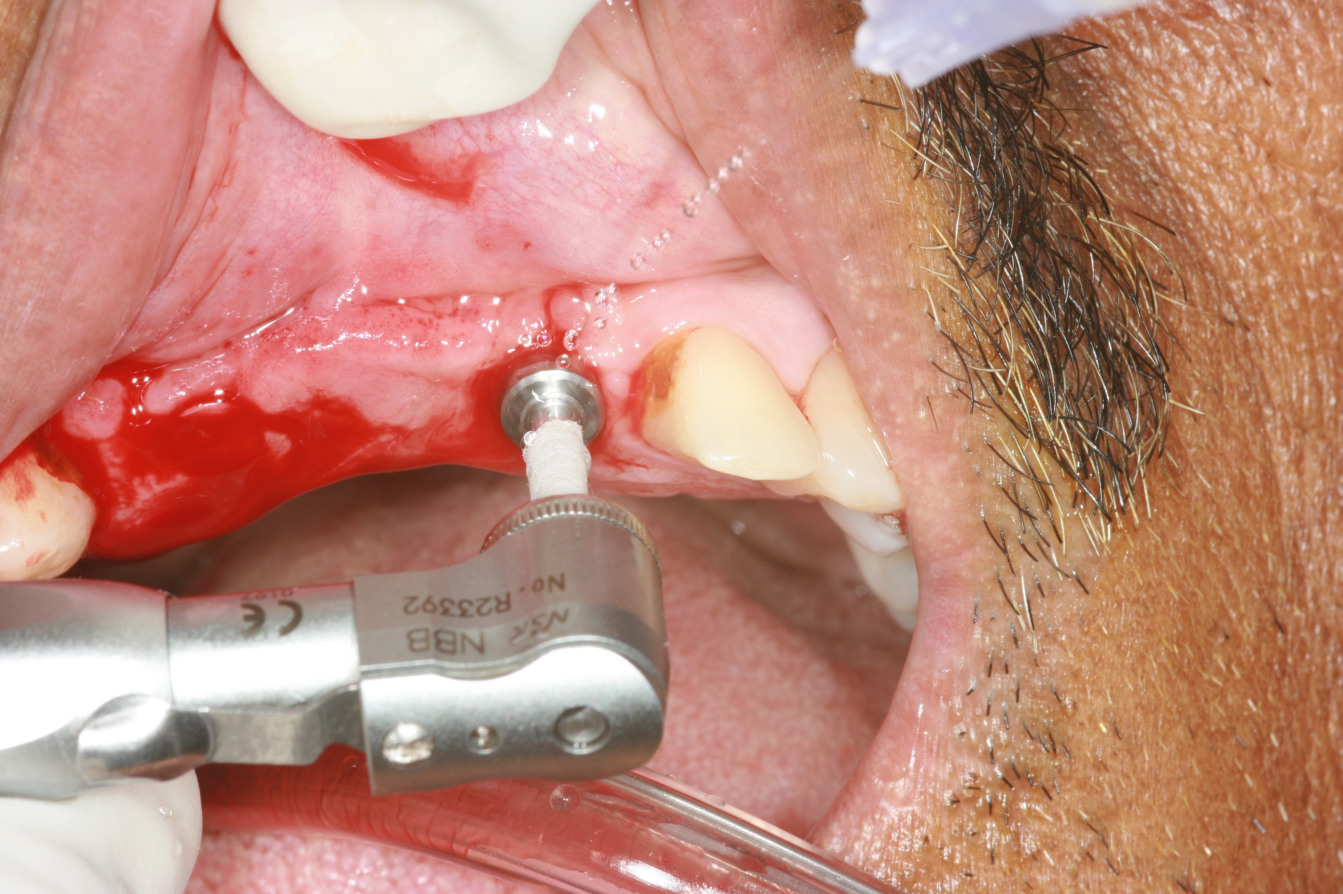
Tissue punch on the predetermined implant site

**Figure 7:**
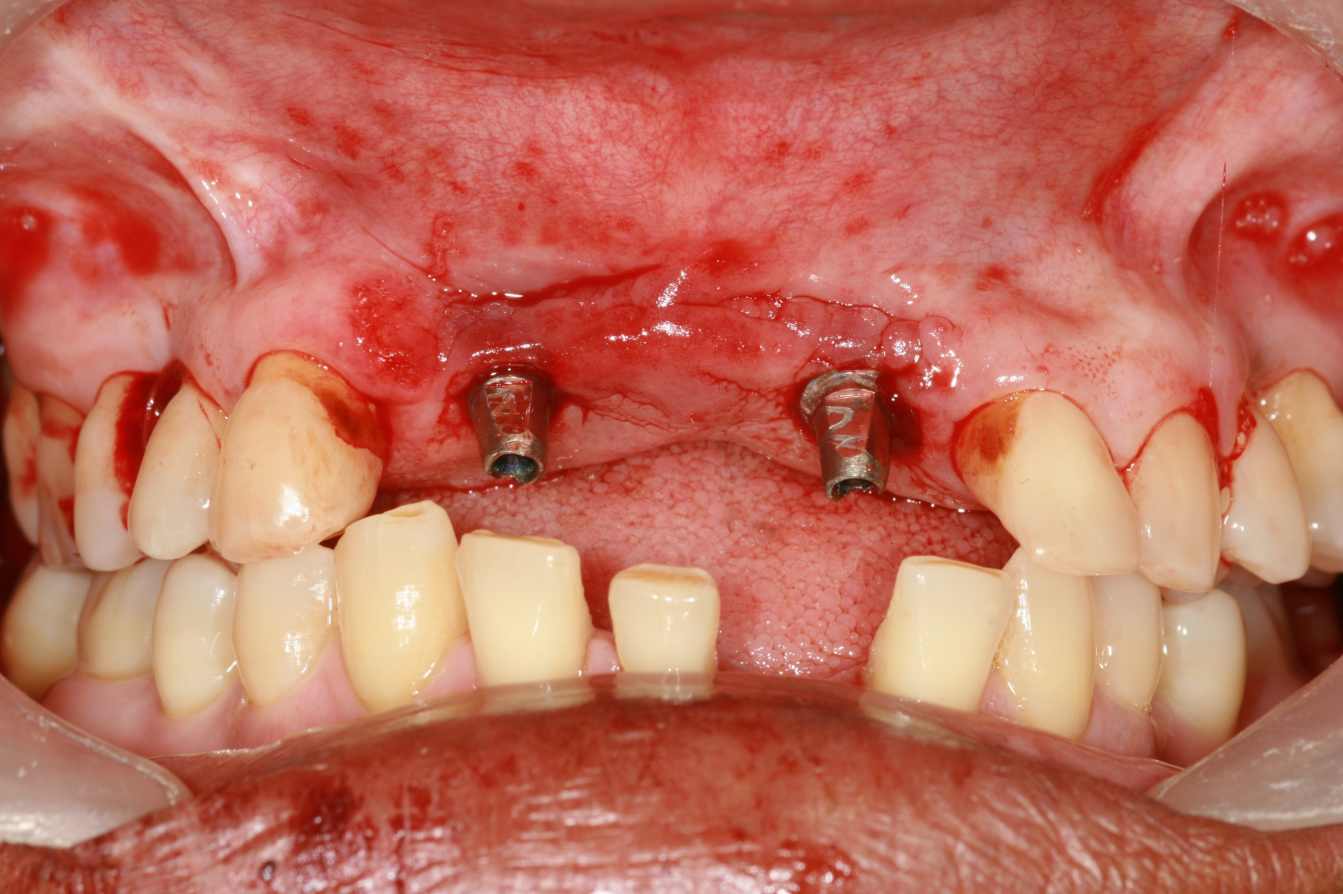
Implant torqued in the osteotomy site

Tin foil was adapted over the abutment as the separating media and chemical cure polymethyl methacrylate (DPI-RR Cold Cure, Dental Products of India, Mumbai) was used for fabrication of provisional restoration. On the day of the surgical procedure, the surgical stent was placed in position, osteotomy was performed for the initial drill, tissue punch was made to remove the overlying tissue (Figure 6), and subsequent osteotomy was performed using the surgical stent. Implants (Uniti implant 3.8-mm width by 13-mm length) (Equinox Medical Technologies B.V., the Netherlands) were torqued, and the prepared abutments were placed in position of 12 and 22 (Figure 7).

**Figure 8:**
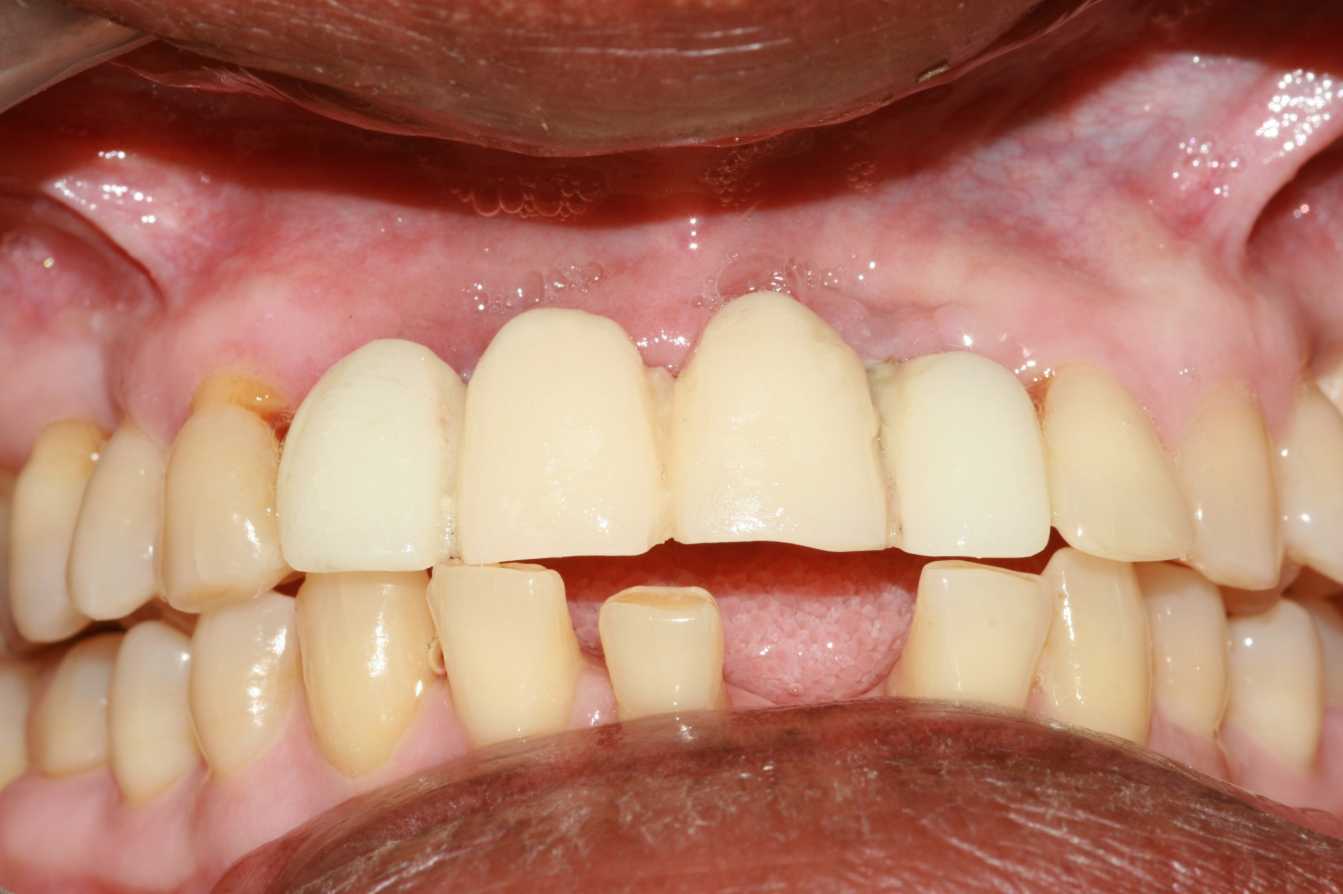
Cementation of provisional intermediate restoration

The intermediate provisional prosthesis was fitted and checked for centric and eccentric contacts and luted with zinc oxide non-eugenol paste (RelyX™ Temp NE Zinc Oxide Non-Eugenol Temporary Cement) (3M, MN, USA) (Figure 8).

Since the angulation of the implant was predetermined, the fitting of the provisional prosthesis did not require any adjustment.

## Discussion

Flapless surgery and immediately loaded implant are gaining popularity with increased patient demand due to reduced patient morbidity. The maintenance of blood circulation and the preservation of gingival architecture are added advantages of flapless surgical procedure [7]. In medically compromised patients with compromised bone quality and quantity combined with congenital or anti-coagulant-induced bleeding diathesis, the technique of flapless implant placement is more successful due to the transgingival approach. Since the soft tissue being excised is only the size of the implant diameter being used, it acts as a plug that prevents bleeding from the bone and soft tissue [7]. Damage to the lateral wall of the bone is possible due to non-visualization of the alveolar bone in flapless implant surgery; hence, it is better to assess the bone architecture and plan before the flapless implant surgery. Recent studies have reported successful results with the flapless approach, which tested the transfer accuracy of drilling templates using various methods [8]. Therefore, in this present technique, the mock surgical procedure was done in the cast, involving the surgical template in precise position. Presurgical diagnostics with appropriate software programs provides all the information necessary regarding the implant site and anatomical landmarks [9]. With an adequate and precise surgical guide, efficient surgery without bone perforation was possible in the presented technique. The surgical technique with immediate loading of implant using flapless surgery has been discussed previously by several authors; however, the immediate loading of implant required a waiting period for the patient until the lab procedure was completed. Moreover, the preparation of an abutment at the surgical table is not recommended as it induces micromotion and generation of heat.

In the technique explained, the abutment preparation and fabrication of provisional restoration done in the presurgical phase avoided the heat generation and micromotion, which could affect the implant stability. This enabled the immediate loading of the implant on the day of surgery, while maintaining the gingival esthetics using flapless implant surgery. Immediate loading for patients with missing maxillary anterior tooth reduced the overall treatment time, eliminated the second stage surgery, and the agony of  wearing removable prostheses until the healing phase [10]. According to Gapski, et al. in 2003 and Trisi, et al. 2009, immediate loading induces micromotion and instability of the implant [11]. In the presented technique, since the replacement site was the anterior teeth, there is no loading with the opposing dentition in both protrusive and lateral movements with an added advantage of avoiding abutment preparation and impression procedure. However, the probability of forces from lip, tongue pressure, and contact with food are unavoidable; hence, non-occlusal immediate loading was preferred for our technique [12]. The patient gets the prosthesis on the day of surgical procedure with negligent waiting period on post-implant placement.

## Conclusions

The presented technique avoids the abutment preparation and impression procedure, which could be traumatic on the day of surgical procedure. With this method, a patient can leave the surgical table with the prosthesis with minimal trauma and good esthetics.
